# The roles of reactive oxygen species and antioxidants in cryopreservation

**DOI:** 10.1042/BSR20191601

**Published:** 2019-08-28

**Authors:** Jia Soon Len, Wen Shuo Darius Koh, Shi-Xiong Tan

**Affiliations:** Diploma in Biomedical Science, School of Applied Science, Republic Polytechnic, 9 Woodlands Avenue 9, Singapore 738964

**Keywords:** antioxidant, Cryopreservation, oxidative stress, reactive oxygen species

## Abstract

Cryopreservation has facilitated advancement of biological research by allowing the storage of cells over prolonged periods of time. While cryopreservation at extremely low temperatures would render cells metabolically inactive, cells suffer insults during the freezing and thawing process. Among such insults, the generation of supra-physiological levels of reactive oxygen species (ROS) could impair cellular functions and survival. Antioxidants are potential additives that were reported to partially or completely reverse freeze-thaw stress-associated impairments. This review aims to discuss the potential sources of cryopreservation-induced ROS and the effectiveness of antioxidant administration when used individually or in combination.

## Introduction

The ability to keep an organism alive while frozen and allowing it to survive for a prolonged period of time may sound like a scene lifted directly from a science fiction movie. Although freezing complex multicellular organisms remains challenging and often faced significant obstacles during the revival of the frozen organism [1–4], reviving single-cell organisms after a prolonged period of time has been a reality for several decades. Among many cases, the ability to revive single cell prokaryotic organisms such as *Escherichia coli* and *Treponema pallidum* were demonstrated in 1913 and 1954 respectively [5,6]. Such results were also obtained from the unicellular eukaryotic organism *Saccharomyces cerevisiae in* 1902 [7].

In the field of research involving mammalian cells, significant progress was made when Polge et al. [8] successfully revived frozen fowl spermatozoa in 1949 and *Bos taurus* spermatozoa cells in 1952 using glycerol as a cryoprotective agent (CPA) [9]. The subsequent use of dimethyl sulfoxide (DMSO) as CPA, which remarkably preserved erythrocytes, was first reported in the 1950s [10] and is now a common component of cryopreservation medium. Although the ability to allow cells to be transported across the world has fostered trans-global scientific collaborations as well as independent verifications of experimental results and clinical advancements, it is frequently taken for granted. One could only imagine the hindrance to scientific advancements if the cryopreservation techniques were absent. For the past few decades, although significant advancements were made in the cryopreservation field on mammalian cells, the technique is far from perfect. Many researchers face challenges such as poor recovery [11,12], loss of functional characteristics of specific cell types [4,13,14] and, in the case of stem cell research, the inability to retain pluripotency [15,16]. In this review, we focus on the role of reactive oxygen species (ROS), a product of cellular metabolism that can be damaging to cells and how ROS contributes to the undesirable results seen after cryopreservation. We further explore current advancements in using antioxidants to negate these undesirable effects observed in cryopreservation.

## Cryopreservation and ROS production

**Figure 1:**
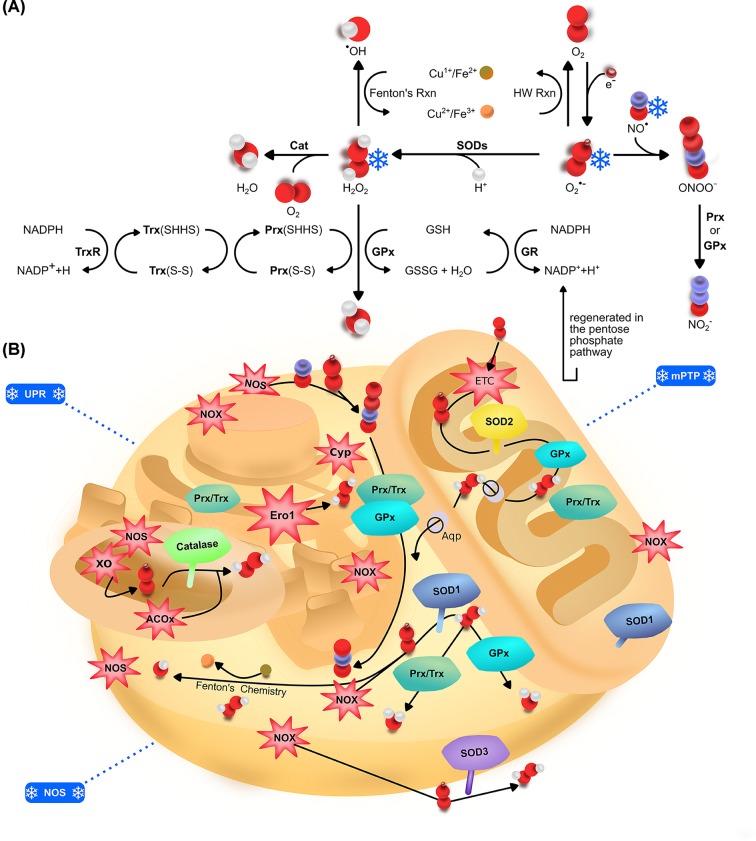
Metabolism and sources of ROS (**A**) Detoxification and metabolism of reactive oxygen/nitrogen species. (**B**) Sources of ROS, and localization of enzymes that counteracts ROS in the mitochondria, endoplasmic reticulum (ER), peroxisome, cytosol and the extracellular space. SOD1 is localized in both the mitochondria intermembrane space and cytosol, SOD3 is located extracellularly and SOD2 is found exclusively mostly in the mitochondria matrix. Catalase that reduces hydrogen peroxide (H_2_O_2_) into H_2_O is mostly located in the peroxisomes. Glutathione peroxidase (GPx) is found in the mitochondria and cytosol. Peroxiredoxins (Prx) and thioredoxins (Trx) which constitute the Peroxiredoxin–Thioredoxin (Prx/Trx) system can be found in the nucleus, mitochondria, ER, peroxisome and the extracellular environment. Electron transport chain (ETC), Cytochrome P450 family of enzymes (Cyps), xanthene oxidase (XO) and NADPH oxidases (NOX) are potential sources of O_2_^•−^, while ERO1 and acetyl CoA oxidases (AcoX) produce H_2_O_2_. Nitric oxide synthase (NOS) is a potential source of NO^•^. Aquaporins (Aqp) facilitate the movement of H_2_O_2_ across membranes. Single snowflake indicates ROS detected while two snowflakes indicate an implication with cryopreservation. Cu^2+^/Fe^3+^ (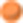); Cu^1+^/Fe^2+^ (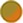); Source of ROS (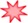); Enzyme (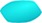); O_2_^•−^ (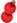); O_2_ (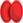); H_2_O (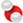); H_2_O_2_ (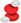); •OH (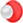); ONOO^−^ (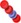); NO^•^ (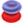); NO_2_^−^ (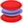); H^+^ (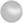); Detected during cryopreservation (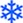); Implicated during cryopreservation (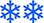).

**Figure 2:**
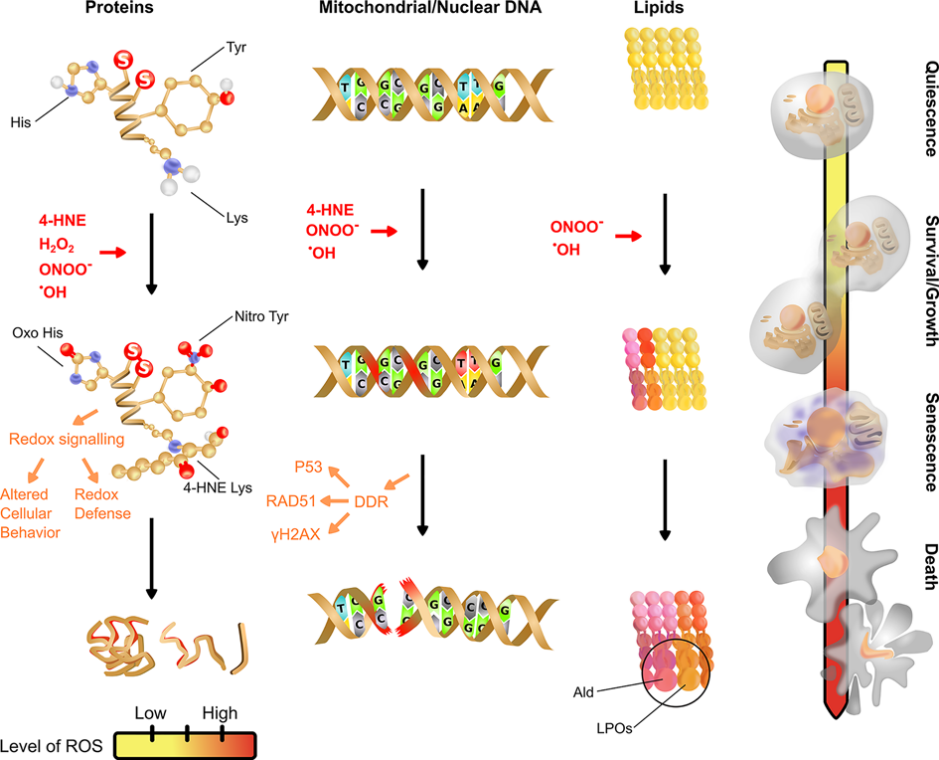
Effects of different levels of reactive oxygen/nitrogen species on cellular biomolecules Protein can react with ONOO^−^, H_2_O_2_, NO^•^, ^•^OH and aldehydes such as 4-Hydroxynonenal (4-HNE) can react with protein side chains (e.g. amino acids such as lysine). The formation of oxo-histidine and disulfide bonds are mostly reversible and mediate redox signaling under mild oxidative stress and may not be deleterious. High level of ROS lead to protein aggregation, denaturation and fragmentation. Mitochondrial/nuclear DNA can react with O_2_^•−^, ONOO^−^ and ^•^OH. Mutations and double/single-strand breaks mediated by ROS are minimized by the DNA-Damage Response (DDR). Proteins such as p53, RAD51 and yH2AX are DDR constituents involved in cryopreservation. Severe oxidative stress can overwhelm the DDR, resulting in mutations and double/single strand breaks. Lipids can react with ONOO^−^ and ^•^OH to cause lipid peroxidation and form lipid peroxides (LPOs). LPOs can decompose into aldehydes (Ald) such as 4-HNE and malondialdehyde (MDA). At low levels of ROS, cells are quiescent. Moderate levels of ROS facilitates beneficial redox signaling to modulate cellular survival, growth and division. Overwhelming levels of ROS can initiate cell death.

Cells have mechanisms to detoxify ROS and once these mechanisms are overwhelmed, ROS can affect various cellular functions and processes by oxidizing proteins, inducing damage to nucleic acids, and peroxidation of lipids [17,18]. Oxidative stress, which is the shift of redox homeostasis toward favoring formation of ROS, dictate the subsequent cellular outcomes such as cellular senescence, apoptosis and altered cellular signaling. Generally, it is known that ROS can modulate cellular survival at low concentrations and death at supraphysiological levels [19]. It is also worth noting that physiological amount of ROS can act as signaling molecules for cellular signaling events [20]. To appreciate the impact of ROS in cryopreservation, it is important to understand the different characteristics of ROS produced in cells, the intracellular sources of these ROS and how cells detoxify these damaging species. Detailed reviews on ROS can be found in published review articles [17,18,21–22] and will not be covered in detail in our current review. A short summary of the sources of ROS and enzymes involved in ROS detexofication is provided in Figure 1 and the section below. Sustained oxidative stress has been believed to be linked to senescence – a response to cellular stress [23], with many lines of evidence supporting this [23–26]. The specific effects of the individual reactive species depend on the relative levels within the cell. The effect of these species at different levels and the biological consequences are summarized in Figure 2.

ROS production has been detected in reproductive and non-reproductive cells. ROS in the form of superoxide (O_2_^•−^) which were detected in the cells of various species undergoing cryopreservation can be reduced with the addition of various antioxidants (Tables 1 and 2). O_2_^•−^ is short-lived and does not cross the mitochondrial or lipid membranes readily due to its charge [27,28]. O_2_^•−^ cannot react with most biological molecules in the aqueous environment of the cytoplasm [18]. O_2_^•−^can be converted into hydrogen peroxide (H_2_O_2_) by three known superoxide dismutase (SOD) isoforms; cytosolic-localized SOD1 (Cu, Zn SOD), mitochondrial-localized SOD2 (Mn SOD) and the extracellular SOD3 (Fe SOD). The localization of the SOD isoforms are reviewed in [29]. Significantly elevated levels of O_2_^•−^ and lipid peroxidation were observed in reviving cryopreserved bull spermatozoa [30]. In alpaca sperm, higher levels of O_2_^•−^ were detected as compared with other oxidizing intermediates using fluorescent dyes dihydroethidium (DHE) for O_2_^•−^ and 2′,7′-dichlorodihydrofluorescein diacetate (H_2_DCFDA) for ROS, which were mainly contributed by cells that are propidium iodide negative [31]. This is also seen in human retinal pigment epithelial (hRPE) cells, where apart from an increase in ROS as detected by H_2_DCFDA only, cellular senescence as well as telomere shortening were reported to increase as a result of cryopreservation [32]. Notably, studies have shown that DMSO treatment of human embryonic stem cells (hES) increases O_2_^•−^ by two-folds while the same cells that were thawed after cryopreservation in the presence of DMSO lead to a five-fold increase in the O_2_^•−^. These data indicate that freeze-thaw stress can promote ROS generation [33]. Other known ice nucleation inhibitors such as anti-freezing protein (AFP) and polyethylene glycol (PEG) have also been known to protect against freeze thaw-induced ROS generation [34–36]. O_2_^•−^ participates in Fenton and Haber–Weiss (HW) reaction in the presence of a ferrous iron catalyst to generate hydroxyl radical (^•^OH) (Figure 1). ^•^OH, unlike O_2_^•−^, can function in the aqueous state and is particularly reactive. ^•^OH is considered the most damaging member of ROS [37] and was reported to cause oxidation of amino acids [38], and these can result in the fragmentation and disruption of protein conformation [39]. ^•^OH can abstract hydrogen and lead to altered nucleic acid bases resulting in DNA damage [40]. Intriguingly, there are no known enzymes to detoxify ^•^OH, despite the damage that it can cause to the cell.

**Table 1:** Antioxidants and their effects on cryopreserved reproductive-associated cells/tissues

Compound	Cell type	Beneficial effects	No effect/adverse effects	Cryopreservation method
2,4-dinitrophenol (DNP)	Sperm	• Motility (↑) [76]^∧^	• Motility(N/C) [76]^∧^	1 cm styrofoam boat on LN at 10 min [76]
Ascorbic acid	Sperm	• ROS^a^ (↓) [149] • Viability (↑) [149] • Motility (Weak ↑) [149] • MMP (↑) [149] • Apoptotic cells (↓) [149] • DNA damage (↓) [148,150]^∧^ [149]	• Motility(N/C) [148]^∧^ • Viability (N/C) [148]^∧^ • DNA fragmentation (N/C or ↑) [148]^∧^ [150]^∧^	LN vapor phase (6.5-2 cm) at 10–15 min [148] LN vapor phase (10 cm) at 10 min [149], −20°C at 10 min + LN vapor phase at 2 h [150]
	Mouse embryos	• Percentage of intact embryos, blastocyst and number of hatching blastocyst (↑) [183] • Number of implantation sites (↑) [183]	• Fetal development (N/C) [183]	Vitrification and slow freezing [183]
Antifreeze proteins (AFP)	Oocytes	• ROS^a^ (↓) [35] • γH2AX+ cells (↓) [35] • Viability (↑) [35] • Cleavage rate, blastocyst rate, blastomere count (↑) [35] • Apoptotic blastomeres (↓) [35] • Improved chromosomal alignment and spindle organization [35]	• Mitochondrial activity (N/C) [35] • Cells with DNA repair (N/C) [35]	Vitrification [35]
BHT	Sperm	• % Motility and viability (↑) [203] • MDA levels^t^ (↓) [203]	• % Sperm with functional intact membrane and GPx activity (N/C) [203]	LN vapor phase (4 cm) at 15 min [203]
Catalase	Sperm	• ROS^a^ (↓) [149] • Viability (↑), weak (↑) motility, MMP [149] • (↓) Apoptotic cells [149] • (↓) Apoptotic like changes, apoptotic and necrotic cells [244] • (↓) DNA damage [149] • Motility (↑) [76]^∧^ • Total and progressive motility, viability, % sperm with high MMP(↑) [244]	• % cells with intact membrane, H_2_O_2_ levels^n^, motility, NO^•^ levels^p^, free iron concentration and functional membrane (N/C) [125] • Motility (N/C) [76]^∧^	LN vapor phase (10 cm) at 10 min [149] LN vapor phase at 20 min [125] 1 cm styrofoam boat on liquid nitrogen at 10 min [76]
	Oocytes	• N.A [124]	• Oocyte survival and fertility (N/C) [124]	Controlled rate freezing [124]
Coenzyme Q	Sperm	• (↑) viability, % sperm with functional membrane and active mitochondria [154] • Weak (↓) number of abnormal cells [154] • Lipid peroxidation [154]^t^ [218]^f (PI staining was done)^ and DNA fragmentation (↓) [154] • (↑) total and progressive motility, plasma membrane integrity [218]^∧^	• Slight (↑) or (N/C) ROS ^k (PI staining was done)^ [218]∧ • O_2_^•− j (stained in association with Yo-Pro® and MitoStatusRed)^ (N/C) [218] • Motility parameters, plasma membrane integrity, MMP and non-capacitated sperm (N/C) [218]^∧^	LN vapor phase (5 cm) at 12 min [154] LN vapor phase (6 cm) at 20 min [218]
Egg yolk	Sperm	• N.A [116]	• (↓) NO^•d^ [116]	LN vapor phase (4 cm) at 10 min [116]
Glutathione (GSH)	Sperm	• Fertilization rate and % cells with ability to undergo acrosome reaction (↑) [41] • Lipid peroxidation^g^, mitochondrial ROS^q^, total ROS^s^ and intracellular ROS^r^(↓) [41] • Motility recovery rate (↑) [153] • % sperm with high MMP, viability, total and Progressive motility(↑) [244] • Apoptotic like changes, apoptosis and necrosis (↓) [244] • Sperm DNA damage (↓) [153]	• Motility-associated parameters (↓) or (N/C) [41] • DNA fragmentation (N/C) [244]	LN vapor phase (N/I) [41] LN vapor phase at 10 min [153]
	Germ cells enriched with spermatogonial stem cells	• N.A [178]	• ATP (N/C) [178] • Proliferation (N/C) [178]	Slow freeze [178]
Hemoglobin (Hb)	Oocyte	• Survival and fertility (↑) [124]	N.A [124]	Controlled freezing [124]
Hypotaurine/Taurine	Germ cells enriched with spermatogonial stem cells	• Proliferation rate and mitochondrial activity (↑) [178]	• Recovery of cells (N/C) [178]	Slow-freeze [178]
	Sperm	• DNA fragmentation (↓) [148]	• Motility and viability [N/C) [148]	LN vapor phase (6.5-2 cm) at 10–15 min [148]
Iodixanol	Sperm	• (↑) motility [200,168], protamine, *BCL2, protamine2/3* and *SPACA3* expression [200] • (↓) *BAX* and *ROMO1* expression and cellular death [200] • (↓) MDA^t^ [168] • (↑) total antioxidant capacity, membrane integrity [168]	• Acrosomal integrity(N/C) [168]	Controlled freezing [168] LN vapor phase (2 cm) at 15 min [200]
L-carnitine	Sperm	• (↑) viability and motility [147]	• DNA oxidation^e^ (N/C) [147]	−20°C at 8 min + LN vapor phase at 2 h [147]
L-proline	Oocyte	• Survival rate (%) (↑) [217] • MMP (↑) [217] • (↓) ROS levels^a^ [217]	• Developmental parameters. apoptosis levels, spindle recovery (N/C) [217]	Vitrification [217]
Lactoferrin/apotransferrin	Mouse embryos	• (↑) percentage of intact embryos and blastocysts [183]	• Hatching blastocyst (N/C) [183]	Vitrification or slow freeze [183]
	Sperm	• (↓) Fe^3+^, NO_2_^− p^ (Griess reagent system) [125] • (↑) percentage of cells with functional plasma membrane [125]	• H_2_O_2_^n^, membrane intactness, motility (N/C) [125]	LN vapor phase at 20 min [125]
Melatonin	Sperm	• (↑) total antioxidant capacity, GSH concentration, functional plasma membrane cells, mitochondrial membrane integrity [199] • (↑) acrosomal integrity [199,240,241] • (↑) MMP [199,240] • (↑) SOD, catalase and GPx activity [199,240,241] • (↑) *BCL-2, SOD2, GSTM1, NRF2, HSP90AA1, catalase* and *HO-1* gene expression [199] • (↓) Lipid peroxidation [199]^u^ [240]^f^ [241]^u^ and ROS [199]^b^ levels [240]^a^ [241] ^u^ • (↓) NADPH oxidase activity [199] • (↓) *Nox5* and *Bax* expression [199] • (↑) Viability [199,156] • (↑) motility [199,156,240,241] • (↑) ATP [156] • (↓) cleaved caspase 3 and 9 [240] • (↓) DNA damage [156] • (↑) 26hpf cleavage rate [156] • (↑) *Bcl2l1* (Bcl-xL) expression and motility [198]	• DNA fragmentation and LDH activity (N/C) [199] • Total blastocyst output (N/C) [156] • Viability, *Bax* expression and ROS^b^ (N/C) [198]	LN vapor phase (10 cm) at 1 h [199] Frozen in pellet-form on dry ice LN vapor phase at 10 min [198] (N/I) Stored in liquid nitrogen [240] Pellet freezing in LN [241]
	Oocytes	(↓) ROS levels^u^, DNA fragmentation and apoptotic gene expression [243] • (↑) expression of telomere maintenance genes [243] • (↑) embryonic stem cell derivation and implantation rate [243]	N.A [243]	Vitrification [243]
MitoTEMPO	Sperm	• (↑) motility, membrane integrity, sperm vitality, MMP, SOD activity, catalase activity, GPx activity, GPI protein levels [214] • (↓) MDA levels^t^ [214]	• Reversal of some beneficial effects (at 500 μM) [214]	LN vapor phase (1–5 cm) at 30 min [214]
Monothioglycerol (MTG)	Sperm	• Mitochondrial ROS^q^ and total ROS^s^ (↓) [41] • Fertility and % cells with ability to undergo acrosome reaction (↑) [41] • Lipid peroxidation^g^ (↓) [41] • (↑) motility recovery rate [153] • (↓) sperm DNA damage [153]	• Motility parameters (N/C) [41]	LN vapor phase (N/I) [41] LN vapor phase at 10 min [153]
NG-nitro-L-arginine methyl ester (l-NAME)	Oocyte	• (↑) fertility and survival (low concentration) [124]	• (↓) Fertility and survival (high concentrations) [124]	Controlled freezing [124]
Quercetin	Sperm	• DNA fragmentation (↓) [153] • Motility and recovery rate (↑) [153] • (↓) % high MMP cells [216]	• Progressive motility, acrosome and sperm plasma membrane integrity (N/C) [216] • (↓) sperm motility recovery rate (at 100 μM) [153]	Controlled freezing [216] LN vapor phase at 10 min [153]
Resveratrol	Sperm	• DNA damage [150], MDA^t^ levels [219] and % high MMP cells (↓) [216] • SOD activity (↑) [219]^∧^	• Progressive motility, acrosome integrity, integrity of sperm plasma membrane (N/C) [216] • Motility (↓) [219] • SOD and catalase activity (N/C) [219]^∧^	Slow cool (−20°C) at 10 min followed by LN vapor phase at 2 h [150] Controlled freezing [216] Slow cool (−20°C) at 10 min followed by LN vapor phase(N/I) at 2 h [219]
SOD	Oocyte	• (↑) fertility and survival [124]	• Decrease in fertility [124] (low concentration)	Controlled rate freezing [124]
	Sperm	• (↑) motility [76]^∧^ • (↓) Reduced apoptotic like changes [244]	• Motility (N/C) [76]∧ • Total and progressive motility, DNA fragmentation, viability, % sperm with high MMP (N/C) [244] • Increased late apoptotic and necrotic cells [244]	1 cm styrofoam boat on LN at 10 min [76]
Trehalose	Germ cells enriched with spermatogonial stem cells	• (↑) proliferation, recovery of colonies after culture and cell viability [207] • Apoptosis (↓) [207]	• Formation of colonies after transplantation (N/C) [207]	Slow freeze [207]
	Testicular tissue	• (↑) cell viability, GSH content and T-AOC [204] • (↓) Lipid peroxidation^t^ [204] • (↑) SOD and catalase activity [204]	• N.A [204]	−20°C at 2 h, −80°C at 12 h [204]
Vitamin E	Sperm	• (↑) Motility [76] • (↓) DNA fragmentation^∧^ [148] • MDA (↓)^t^ [215]	• Viability and motility (N.C) [148] • Motility (N/C) [215] • O_2_^•−^ production in live cells^i^ (N/C) [215]	[Controlled rate freezing] 62.3°C/min [215] 1 cm styrofoam boat on LN at 10 min [76] LN vapor phase (6.5-2 cm) at 10–15 min [148]
Zinc oxide nanoparticles	Sperm	• DNA damage and lipid peroxidation^t^ (↓) [155]	• Sperm motility and ability to undergo the acrosome reaction (N/C) [155]	(N/I) Stored at −196°C [155]
Zinc sulfate	Sperm	• DNA damage (↓) [220] • (↑) Mitochondria integrity, % sperm with ability to undergo acrosome reaction and capacitation [220] • (↑) Motility (↑) [220]^∧^	• Motility (N/C) [220]^∧^	LN vapor phase at 5 min [220]
Trolox (Vitamin E analog)	Ovarian tissue	Viable follicles (↑) [81] • *BMP4, BMP15, CTGF, GDF9, KL* expression (↑) [81] • Trolox equivalent antioxidant capacity values (↑). [81]	*HSP70, ERp60, SOD1* and *ERp29*, *AMH* expression (N/C) [81]	2°C/min from 20 to −7°C; cooled at 0.3°C/min to −30°C, into LN (−196°C) [81]

Abbreviations: BHT, butylated hydroxytoluene; LN, liquid nitrogen; MDA, malondialdehyde; MMP, mitochondrial membrane potential; N.A, not-applicable; N/C, no changes/no effect. -, no effects have been reported. (↑) and (↓), represent a significant increase or decrease respectively. ^∧^, denotes cases where effects are context dependent and due to factors such as cell quality and species. Method employed for detection of ROS and Oxidative biomarkers are denoted by alphabetical superscripts ‘a’ to ‘u’: a, H_2_DCFDA. b, H_2_DCFDA/Propidium Iodide (Pi). c, 4,5-diaminofluorescein diacetate (DAF-2DA). d, DAF-2DA/Ethidium Homodimer -. e, 8-OHG. f, BODIPY 581/591 C11. g, BODIPY 581/591 C11/Propidium Iodide (Pi)-. h, Bromopyrogallol Red. i, DHE/Sytox-. j, DHE. k, Dihydrorhodamine(DHR) 123/Propidium Iodide (Pi)-. l, DHR 123. m, 2,4-dinitrophenylhydrazine (DNPH) assay. n, Fox2-modified method. o, Formamidopyrimidine-DNA glycosylase-sensitive comet assay. p, Griess reagent system. q, MitoPY1/SYTOX–. r, PF6-AM/SYTOX. s, Peroxy Green 1 (PG1). t, Thiobarbituric acid reactive substances (TBARS) assay. u, commercial or obscure ROS detection techniques.

**Table 2:** Antioxidants and their effects on non-reproductive cell types/tissues

Compound	Cell type	Beneficial effects	No effect/adverse effects	Cryopreservation method
Ascorbic acid	Bone-marrow mononuclear cells	• Clonogenic parameters (↑) [221] (murine model)	• Viability and clonogenic parameters (human model) (N/C) [221]	Controlled rate freezing [221]
Astragalosides	Pancreatic islets	• Restored blood glucose to normal [232] • Insulin expression after transplantation (↑) [232]	• N.A [232]	Slow-freeze [232]
BHT	Blood cells	• Loss of HUFAs (↓) [184]	• N.A [184]	Chromatography paper at −20°C [184]
BHT + ascorbic acid	Hepatocytes	• Post-thaw albumin production (↑) [235]	• Induced LDH release (↑) [235] • Urea synthesis, ammonia clearance and cell proliferation (N/C) [235] • Apoptosis associated DNA fragmentation (N/C) [235]	(N/I) stored in −70°C freezer [235]
Catalase	Mononuclear cells	• Clonogenic parameters (↑) (murine model) [221]	• Viability, clonogenic parameters (human model) (N/C) [221]	Controlled rate freezing [221]
Catalase + Trehalose	Hematopoietic cells	• DCF fluorescence intensity^a^ (↓) [233] • Number of DCF+ cells^a^ (↓) [233] • (↑) CFU [233,234] • (↑) pre-CFU [233] • Better engraftment [233] • (↑) viability [233] • Apoptosis (↓) [233] • (↑) responsiveness to migratory homing associated cytokines, expression of homing-associated receptor and adhesion capacity [234]	• N.A [233,234]	Controlled rate freezing [233,234]
Consumption of blueberries by PBMC donors	Peripheral blood mononuclear cell	• DNA oxidation (↓)^o^ [144]	• DNA damage induced by H_2_O_2_ in cryopreserved cells (N/C) [144]	Slow freeze [144]
Deferoxamine	Blood cells	• Loss of HUFA (↓) [184]	• N.A [184]	Chromatography paper at −20°C [184]
Glutathione (GSH)	Embryonic stem cells	• ROS^a^ (↓) [247] • Viability (↑) [247]	• N.A [247]	(N/I) Stored in −80°C freezer at 24 h [247]
	Embryogenic callus	• Post-thaw survival, GSH, ascorbic acid levels, SOD and peroxidase activity (↑) [174] • ^•^OH^h^, H_2_O_2_^u^, O_2_^•−u^ and MDA levels^t^ (↓) [174]	• At high concentrations, survival (↓) or (N/C) [174] •catalase activity (N/C) [174]	Vitrification [174]
	Pancreatic islets	• MDA^t^ (↓) [222] • Islet morphometry and glucose clearance rate (↑) [222]	• Islet insulin secretion (N/C) [222]	Slow freeze [222]
Peroxiredoxin	Murine hepatocytes	• Viability (↑) [236] • Integrin-β1 and β-catenin cell adhesion proteins (↑) [236] • Urea secretion (↑) [236] • NO^•c^(↓) [236] • ROS^a _^(↓) [236] • O_2_^•−j^(↓) [236]	• E-Cadherin cell adhesion proteins (N/C) [236]	Slow freeze [236]
	Murine insulinoma	• Viability (↑) [236] • Insulin secretion (↑) [236] • NO^•c^ (↓) [236] • O_2_^•−j^ (↓) [236]	• ROS^a^ (N/C) [236]	Slow freeze [236]
Polyethylene glycol (PEG)	Human embryonic stem cells	• (↓) ROS^j^ [36] • Alleviation of F-actin levels [36]	• Cell viability (N/C) [36]	Slow freeze [36]
S-Adenosylmethionine	Hepatocytes	• (↑) GSH content and cellular viability [177]	• N.A [177]	Slow freeze [177]
Salidroside	Red blood cell	• (↓) protein carboxylation^m^ [158] • (↓) Lipid peroxidation^t^ (when trehalose was used as a CPA) [158]	• Lipid peroxidation^t^ (N/C) (when used with glycerol as a CPA) [158]	N.I [158]
SOD	Bone-marrow mononuclear cells	• N.A [221]	• Post-thaw recovery (N/C) [221]	Controlled rate freezing [221]
Trehalose	Dendritic cells	• Preserved cell function and phenotype [205] • (↑) viability [205] • Maintained MMP and cytoskeleton integrity [205] • (↓) apoptosis, *BIM-1* and CASP9 expression [205]	• N.A [205]	Controlled rate freezing [205]
	Hepatocytes	• (↑) albumin secretion, plating efficiency and viability [206] • (↓) AST activity [206]	• EROD and ECOD activity, proliferation, LDH, urea levels (N/C) [206]	Controlled rate freezing [206]
	BM-MNC	• (↑) Clonogenic parameters (murine and human models [221]	• N.A [221]	Slow controlled rate freezing [221]
Wheat proteins or Lipocalins	Hepatocytes	• (↑) attachment efficiency and viability [227] • Restoration of cytochrome P450 isoform activity to fresh cells levels [227]	• N.A [227]	Slow freeze [227]

Abbreviations: AST, aspartate aminotransferase; CFU, colony forming units; DCF, 2′,7′-dichlorofluorescein; BHT, butylated hydroxytoluene; HUFA;highly unsaturated fatty acid; LN, liquid nitrogen; MDA, malondialdehyde; N.A, not-applicable; N/C, no changes/no effect. -, no effects have been reported. (↑) and (↓), represent a significant increase or decrease respectively. ^∧^, denotes cases where effects are context dependent and due to factors such as cell quality and species. Method employed for detection of ROS and Oxidative biomarkers are denoted by alphabetical superscripts ‘a’ to ‘u’: a, H_2_DCFDA. b, H_2_DCFDA/Propidium Iodide (Pi). c, 4,5-diaminofluorescein diacetate (DAF-2DA). d, DAF-2DA/Ethidium Homodimer-. e, 8-OHG. f, BODIPY 581/591 C11. g, BODIPY 581/591 C11/Propidium Iodide (Pi)-. h, Bromopyrogallol Red. i, DHE/Sytox-. j, DHE. k, Dihydrorhodamine (DHR) 123/Propidium Iodide (Pi)-. l, DHR 123. m, 2,4-dinitrophenylhydrazine (DNPH) assay. n, Fox2 modified method. o, Formamidopyrimidine-DNA glycosylase-sensitive comet assay. p, Griess reagent system. q, MitoPY1/SYTOX–. r, PF6-AM/SYTOX-. s, Peroxy Green 1 (PG1). t, Thiobarbituric acid reactive substances (TBARS) assay. u, Commercial or obscure ROS detection techniques.

With respect to H_2_O_2_ levels in cells during cryopreservation, total H_2_O_2_ levels remain largely unchanged, while mitochondrial H_2_O_2_ were reported to be increased in spermatozoa [30,41]. H_2_O_2_ has highly selective reactivity with only certain biomolecules and can cause the oxidation of thiol groups (SH). H_2_O_2_ is toxic at high concentrations because it can be reduced by ferrous Iron, Fe (II), into the more damaging ^•^OH via the Fenton reaction (Figure 1) [42–44]. H_2_O_2_ has a long half-life which allows it to transduce signals at long ranges [37,45]. When in extracellular space, H_2_O_2_ can re-enter the cell through aquaporin-dependent pathways or via direct diffusion [46]. Multiple pathways such as the glutathione peroxidase-glutathione reductase and the peroxiredoxin/thioredoxin-thioredoxin reductase pathways utilize NADPH as reducing equivalent to reduce H_2_O_2_ to H_2_O (Figure 1). The species of ROS and methods used for detection in different cell types used for cryopreservation are summarized in Tables 1 and 2.

### Mitochondrial ROS production

Studies from fish [47], sheep [48] and human cells [49] have indicated that cryopreservation induced alterations and/or damages to the mitochondria. Proteins upstream in the electron transport chain (ETC) can generate ROS through the univalent donation of electrons to oxygen in the mitochondria. Sources of ROS in the mitochondria include complex I, complex II and complex III [50]. These enzymes ‘leak’ electrons and as a result, univalently reduce oxygen to O_2_^•−^. Through this process, ROS in the form of O_2_^•−^, ^•^OH and H_2_O_2_ are produced (Figure 1).

Factors influencing the production of ROS in the mitochondria include: tissue or cell type, oxygen tension of the extracellular environment, presence of metabolic intermediates and substrates [51], hyperoxia [50,52], the presence of a high proportion of NADH electron donors [51,53] as well as the mitochondrial membrane potential (Δψ) and the pH gradient [54–56], which are constituents of proton-motive force, Δp. The multitude of mitochondria inducers underlie the fact that multiple mechanisms can affect the genesis of mitochondrial ROS in the ETC (reviewed in [50,51,56]).

The MMP or Δψ is a parameter widely used to assess mitochondrial function. Δψ was reported to be altered in thawed cells following cryopreservation [57–59]. Reduction in Δψ in certain cases, such as a mild decrease, is associated with a decline in ROS levels while an increase in Δψ has been noted to promote ROS formation in rat mitochondria isolated from brain [60] and heart muscles [61]. These studies indicated that maintenance of the Δψ is an important aspect to prevent ROS-induced oxidative stress during cryopreservation.

Hyper-polarization of the Δψ can favor ROS generation [62], which is believed to be a result of a reduction in electron transfer [63]. Depolarization of Δψ can be induced by ROS, which impairs oxidative phosphorylation and amplifies ROS generation [64]. Loss of Δψ was reported in cryopreserved human oocytes [57], buffalo sperm [65,58], nucleus pulposus-derived mesenchymal stem cells [66], murine embryos [67], *Meleagris gallopavo* spermatozoa [68], koala spermatozoa [69] and porcine hepatocytes [59], although a transient elevation in Δψ was reported in murine oocytes after freeze-thawing [70]. Opening of mtochondrial permeability transition pore (mPTP), which involves the formation of a ‘hole’ in the inner mitochondrial membrane (IMM) is known to lead to the dissipation of the Δψ as well as an elevation in ROS levels [64]. Opening of mPTP leads to dissipation of the Δψ, mitochondrial swelling, ATP depletion, relocalization of pro-apoptotic molecules and elevated ROS levels [71,72].

The involvement of mPTP in cryopreservation has been implicated in the study showing that inhibition of mPTP by bongkrekic acid successfully reduced cryopreservation-induced apoptosis in stallion spermatozoa [73]. mPTP opening has been known to enhance H_2_O_2_ production through conformational alterations to complex I of ETC [74], depletion of ROS-scavengers as well as intensifying production of ROS from Krebs cycle oxidoreductases [75]. Opening of mPTP is known to be induced during oxidative stress and ROS-mediated alterations to mPTP regulators and components were suggested to be responsible for this. Indeed, it was found that the mild uncoupling agent 2,4-dinitrophenol, which normally reduces ROS, improved motility in sperm with low cryopreservability [76] while the antioxidant, monothioglycerol was found to reduce mitochondria ROS as well as increase fertility and the percentage of cells with the ability to undergo acrosome reaction [41]. ROS-mediated mitochondrial permeabilization involved oxidative attack on the protein thiol groups on the mitochondrial membrane. This may give rise to protein aggregates due to thiol groups cross-linking after being oxidized [77,78].

### Protein folding in the endoplasmic reticulum and ROS production

Cryopreservation of cells was known to perturb the homeostasis of the endoplasmic reticulum (ER) [79–82] and ER is a known source of ROS [83,84]. The ER facilitates the proper folding and addition of some post-translational modifications to proteins in the secretory pathway. Accumulation of misfolded proteins in the ER could occur under conditions that perturb ER homeostasis, also known as ER stress. Increased protein synthesis is one such condition. The unfolded protein (UPR) response promotes an adaptive response against ER stress by increasing machineries for protein degradation and protein folding as part of an effort to restore ER homeostasis. The molecular mechanism on how UPR are activated has been comprehensively reviewed by [85,86]. UPR is activated via one of the three membrane-bound transducing receptors (ATF6, PERK, IRE1α), these three sensors thus constitute the three branches of the UPR signaling pathways [85,86]. It was observed that *SOD1* and the ER stress marker *ERP29* gene expression were significantly up-regulated in response to freeze-thaw stress in primate ovarian tissue [81]. In yeast, genes expression for protein chaperones such as *SSA4, HSP26, HSP42* were found to be up-regulated in response to freeze-thaw stress when cells were frozen in the absence of cryoprotectants [87]. In mammals, all three arms of the UPR may be activated during cryopreservation. The XBP-1 protein levels were elevated in vitrified mice oocytes [79] and maturing oocytes exposed to delipidated serum were more susceptible to cryopreservation-induced ER stress [80]. Intriguingly, the handling of oocytes, itself, was sufficient to activate the IRE1α arm [88], highlighting the vulnerability of oocytes to cope with stress during the cryopreservation process.

In addition to its homeostatic role, sustained induction of the UPR in response to severe ER stress caused by a multitude of factors can antagonize cellular survival, resulting in cell death [89]. The activation of the UPR has been implicated in the production of at least two species of ROS: O_2_^•−^ and H_2_O_2_ [90–92] and these ROS were postulated to be an event preceding cellular death. The source of H_2_O_2_ may be attributed to oxidative folding via the protein disulfide isomerase (PDI)-ER oxidoreductase (ERO) relay or the cytochrome P450 (CYP) family of enzymes. The PDI-ERO1 pathway has been demonstrated to produce ROS in the form of H_2_O_2_ [93–95]. Activation of the PERK-arm of the UPR could lead to downstream ERO1α activation, H_2_O_2_ production and mediates the feeding of calcium into the mitochondria which could promote O_2_^•−^ production and apoptosis [92]. Interestingly, a yeast strain deleted for genes encoding for catalases and glutathione were hypersensitive to exogenous H_2_O_2_, but are not sensitive to *ERO1* overexpression [96], indicating that there could be other, more potent sources of H_2_O_2_ in the ER, or that cytosolic or mitochondria pool of H_2_O_2_ are isolated from the ER. Studies have also indicated an ERO1-independent source of H_2_O_2_ in the ER [97,98], suggesting the PDI-ERO1 pathway may not be the sole source of H_2_O_2_ in the ER. Other possible pathway that could be activated through sustained UPR activation that may lead to mitochondrial ROS generation is through the dimerization of IRE1α, which activates the JNK-SAB axis to initiate cellular death [99].

### Nitric oxide synthase and NADPH oxidase

Although not considered ROS, nitric oxide (NO^•^) and peroxynitrite (ONOO^−^) are free radicals. NO^•^ can diffuse through the cell membrane. *In vivo*, NO^•^ is not highly reactive to most biomolecules. However, NO^•^ can react with metal complexes to form metal nitrosyls. NO^•^ reacts with O_2_^•−^ to form more damaging species, such as ONOO^−^ which is thought to occur mostly in the hydrophobic regions of the cell [100]. ONOO^−^ can be detoxified by enzymes such as peroxiredoxins and glutathione peroxidase [101] (Figure 1). Unlike NO^•^, ONOO^−^ are strong oxidants capable of causing oxidative damage, nitration and S-nitrosylation of proteins [102,103]. *In vivo*, nitric oxide (NO^•^) is produced by the family of nitric oxide synthase (NOS) which consist of three isoforms: neuronal NOS or NOS1 (‘neuronal’ NOS/nNOS), NOS2 (‘inducible’ NOS/iNOS) and NOS3 (‘endothelial’ NOS/eNOS). NOS typically catalyzes the formation of NO^•^ and citrulline from arginine and oxygen. Most NOS isoforms are usually regulated by calmodulin and calcium, and require the cofactors NADPH, FAD, Flavin mononucleotide (FMN) and tetrahydrobiopterin (BH4) [104,105]. Similar to ROS, NO^•^ regulates cell death and survival [106–110]. NO^•^ is essential for proper cellular physiological function such as vasodilation [111] as well as regulating immunosuppression [112] and tissue repair in mesenchymal stem cells (MSCs) [113]. Conversely, NO^•^, can interfere with hemopoiesis [114]. Moderate levels of NO^•^ can initiate capacitation [115] and is essential for motile functions in sperm [116,117].

During cryopreservation, NOS activation or NO^•^ production was observed in cryopreserved heart valves [118] and sperm [30,116,119]. While NO^•^ itself has not been found to be significantly increased by freeze-thaw stress in RBC, the product of nitric oxide nitrosylation, S-nitrosohemoglobin was found to be increased by freeze-thaw stress [120]. At high levels of NO^•^, sperm functions can be antagonized [116,121–123]. When cryopreserving sperms, the use of low concentrations of NOS inhibitor, NG-nitro-l-arginine methyl ester (l-NAME) [124], and anti-nitrosative agents such as hemoglobin [124] and lactoferrin [125] has been found to improve membrane functionality, survival and/or fertilization, indicating that reducing NO^•^ may improve assisted reproductive technology outcomes especially since NO^•^ was elevated in post-thawed cells. It should however be noted that the use of high concentrations of l-NAME was found to impair sperm function [124].

NADPH oxidases (NOXs) are a family of seven-membered enzymes that are highly regarded due to their role as a major non-mitochondrial ROS generator. NOX enzymes generate ROS, primarily O_2_^•−^, by catalyzing the transfer of one electron across the membrane from the electron-donating NADPH to the electron acceptor oxygen, thus reducing oxygen to form O_2_^•−^. Exceptions are NOX4, DUOX1 and DUOX2 of the NOX/DUOX family, which was documented to produce mainly H_2_O_2_ [126]. While all seven members of the NOX family are found to be located to the plasma membrane, specific NOX isoforms such as NOX4, NOX5 and DUOX2 have also been detected at ER, with NOX4 residing at other subcellular locations including the mitochondria and the nuclear membrane [126,127]. Apart from the mitochondria and ER, the peroxisome is another source of intracellular ROS, which harbors pro-oxidant enzymes such as acyl CoA oxidase (ACOx) and xanthine oxidases (XOs) [128].

Our current understanding of the activation of NOX includes a collection of inducers which can be sorted into three main categories namely: chemical, biological and physical [129]. With respect to cryopreservation, NOX activation induced by chemical and physical inducers are particularly relevant and worth noting. Physical inducers are a broad collection of inducers including temperature [130], osmotic stress [131] and pH changes [132] which are documented NOX-inducers, that are coincidentally generated during cryopreservation [133]. During cryopreservation, the addition and removal of cryoprotectants, as well as freeze-thawing have been proven to subject cells to osmotic stress [134]. Extracellular ice formed during cryopreservation puts the cell through hypertonic conditions as the solute concentration elevates in the unfrozen extracellular portions. As a result, cells shrink as water leaves the cell to re-establish the equilibrium of solute concentration across the cell. The reverse is also true for thawing during cryopreservation, in which this time, cells are put through hypotonic conditions which lead to movement of water into the cell, consequently, cell swelling. Swelling of cells under hypotonic condition, however, was viewed as more pernicious due to the elevation in ROS levels following cryopreservation in the case of stallion sperm [135].

In astrocytes, hypo-osmotic swelling leads to an increase in ROS as well as phosphorylation of p47^phox^ and that, apocynin, an NOX inhibitor abrogated such effects [136]. Moreover, cortical brain slices of mice with p47^phox^ knockout failed to show elevated ROS levels as observed in wild-type mice suggesting hypo-osmotic swelling results in p47^phox^-NOX-dependent generation of ROS [136]. In agreement with this, supporting evidence from skeletal muscles, in which osmotic stress leads to localized increase in Ca^2+^ in the cytosol, termed as ‘calcium spark’ and an elevation in ROS levels have further substantiated this viewpoint. In addition to this, treatment of skeletal muscle cells with NOX inhibitors, apocynin and diphenyleneiodonium, reversed this effect. The exclusion of extracellular Ca^2+^ restrained the increase in levels of ROS as well as calcium spark and the inhibition of Ca^2+^ release from the sarcoplasmic reticulum by the inhibitors, ryanodine and thapsigargin were able to further reduce ROS levels [131].

Taken together, the above observations suggested that NOX activation via osmotic stress may be dependent on Ca^2+^ release from the sarcoplasmic reticulum. The Ca^2+^ could then influx into mitochondria from osmotic stress, leading to induction of NOX activity [131]. Thus, it could be inferred from the above studies that cryopreservation may induce NOX activation. Whether NOX inhibitors can abrogate the ROS generated in post-thawed cells remains to be investigated. Current findings indicate that NOX activation during cryopreservation could be a potential target to reduce ROS-induced damage in cells.

## DNA damage, protein oxidation and lipid peroxidation in cryopreservation

Detection of oxidative damage in cryopreserved cells is a valuable measurement to determine the degree of damage. Many consequences of ROS-induced damages can be credited to lipid peroxidation [137], DNA damage [138] and protein oxidation [139,140] (Figure 2). Methods used for measurement of these damages are reviewed by [141].

Cryopreservation significantly increased DNA damage in cells as assessed by the comet assay or DNA fragmentation. Activation of DNA damage repair (DDR) constituents such as p53 [33,142], γH2AX and RAD51 [143] were observed during slow-freeze and/or vitrification. DNA oxidation was increased in cryopreserved human peripheral blood mononuclear cells (PBMCs), indicating oxidative damage has occurred in these cells [144]. In contrast, PBMCs from donors who consumed wild blueberries rich in antioxidants has been reported to have significantly lower DNA oxidation following cryopreservation. Whereas antioxidants may reduce DNA oxidation during freeze-thawing, l-carnitine, an antioxidant [145,146], has however, failed to reduce DNA oxidation in thawed human spermatozoa *in vitro* [147]. Addition of compounds with known antioxidant properties such as vitamin C [148–150], vitamin E [148,151], resveratrol, [150], β-mercaptoethanol [152], taurine, hypotaurine [148], glutathione (GSH) [153], coenzyme Q [154], quercetin [153], zinc oxide nanoparticles [155], catalase [149] monothioglycerol, glutathione [153] and melatonin [156] have been reported to significantly reduce DNA damage in cryopreserved cells (Table 1).

Protein oxidation, as determined by protein carbonylation were detected in cryopreserved cells. Freezing stress was characterized to lead to the formation of carbonyl groups in intact and homogenized tissue [157]. RBCs cryopreserved with glycerol or trehalose were found to have increased ROS accumulation and protein oxidation. Supplementation of the antioxidant Salidroside ameliorated this effect [158]. Protein oxidation and increased ROS was also detected in cryopreserved sperm cells [159]. Lipid peroxidation can be due to the effect of ^•^OH and ONOO^−^ [160,161]. Increase in lipid peroxidation was observed in tissue specimens stored at −20°C [162]. Lipid peroxidation as measured by either malondialdehyde (MDA) or 4-Hydroxynonenal (4-HNE) were detected in cryopreserved red blood cells [158], sperm [163–166] and hepatocytes [167]. Notably, the product of lipid peroxidation 4-HNE is extremely reactive, which allows it to react with DNA and proteins. Antioxidants such as iodixanol can reduce lipid peroxidation in cryopreserved buffalo semen [168].

## Effectiveness of antioxidants in preventing cryoinjury: lesson learnt so far

### Endogenous defense mechanisms and effects of inhibitors on ROS-generating sources in cryopreservation

Transcriptomic studies have shown that many antioxidant genes such as *SOD1*, cytosolic catalase T (*CTT1*) and glutaredoxin-1 (*GRX1*) were induced in the model eukaryote *Saccharomyces cerevisiae*, also commonly known as the baker’s yeast or brewer’s yeast [169]. These studies indicated the importance of the role of antioxidants in mitigating freeze-thaw stress after cryopreservation [169]. Intriguingly, genetic screening of yeast mutants defective for different antioxidant genes highlighted that not all antioxidants contribute equally in their ability to protect cells from freeze-thaw stress [87,170]. It was found that yeast strains deleted for *SOD1* and *SOD2* were particularly sensitive to freeze-thaw stress, while single deletion of catalase and glutathione peroxidase were not as sensitive [170]. In mammals, both vitrification and slow freezing were found to up-regulate SOD gene expression and increase proteins levels in murine oocytes [171], embryos [172] and testicular tissue [142]. Furthermore, the addition of O_2_^•−^ scavenging agent MnCl_2_ rescued cells deleted for the *SOD1* gene [170]. Collectively, these studies highlight the importance of the SOD genes in cryopreservation of various cell types.

Besides SOD, the reduced GSH regeneration system or the pentose-phosphate shunt for NADPH production were up-regulated in *S. cerevisiae* during freeze-thaw [169]. Given that GSH is the most abundant antioxidant in almost all cell types [173], it is therefore not surprising that the glutathione cycle is required for freeze-thaw tolerance. Studies where spermatozoa were administered with GSH or thiols were demonstrated to modestly reduce ROS [174], increase the motility of spermatozoa [41,175] and the developmental competence of mouse oocytes [176]. In addition, the GSH and cysteine precursor, S-adenosylmethionine, increased the total GSH levels and the viability of cryopreserved cells. While the supplementation of S-adenosylmethionine lead to significantly lower MDA levels in cold-stored rat hepatocytes, it was however not determined in the cryopreserved cell group [177]. The proliferation of spermatogonial stem cells was however noted to be unaffected by administration of glutathione [178].

Interestingly, one of the more oxidizing environment in the cell is the ER, where the reduced GSH to oxidized glutathione (GSSG) ratio is 3:1 as compared with the cytosol where the ratio is 100:1 [179]. Perturbation of ER homeostasis was known to trigger the UPR. The induction of UPR coincides with a reduction in the developmental competence and modest reductions in survival of cryopreserved cells, which can be improved by supplementation of ER stress inhibitor TUDCA [79,80,82]. The use of Trolox, a water-soluble analog of vitamin E, increased antioxidant capacity, prevented ER stress and improved the viability of ovarian tissues. This indicates a role for both oxidative stress and ER stress during cryopreservation [81]. Intriguingly, it was found that an inhibitor that prevents ER stress-induced apoptosis, Salubrinal, did not improve development and viability of bovine blastocyst [180], indicating that preventing ER stress-induced cell death alone may not be sufficient to prevent cryopreservation-induced damage. However, it is worthy to note that in this specific case, the viability of the blastocyst is close to 100% in both control and Salubrinal treated groups [180].

Gene expression encoding for proteins which regulate or sequester the availability of Fenton reaction initiators are up-regulated in transcriptomic studies in freeze-tolerant animals or in yeast undergoing freezing stress [181,182]. As antioxidants, iron chelators such as deferoxamine, lactoferrin and transferrin were found to limit NO^•^ production and improve cellular parameters affected by cryopreservation-induced oxidative stress [125,183,184]. Deferoxamine was found to prevent loss of highly unsaturated fatty acids in RBCs stored at −20°C for a shorter period time as compared with the lipophilic free radical scavenger butylated hydroxytoluene (BHT) [184]. Interestingly, supplementation of transferrin, ascorbic acid and a combination of both compounds generally improved the percentage of intact embryos. However only when ascorbic acid was used alone did the number of hatching blastocysts appreciably increase [183]. These studies revealed the complexity of the outcome of cryopreserved cells when using antioxidants as a supplement for cryopreservation.

### Apoptosis as an adversity after cryopreservation

The efficiency of the cryopreservation process is still partially compromised due to several factors. Reduced cell viability, increased senescence and impaired cellular functions are among the most widely reported adversities associated with oxidative stress generated during cryopreservation of cells. In spermatozoa, freeze-thawing during cryopreservation greatly reduced cell viability accompanied by a range of structural abnormalities and damages, presumed or found to be a consequence of oxidative stress [185–187]. Supplementation of antioxidants into the cryopreservation media generally yielded good cell viability, indicating that oxidative stress plays a role in inducing cellular death during cryopreservation [147,188,189]. Cryopreservation led to re-localization of phosphatidylserine from the inner to the outer leaflet of plasma membrane, a signal displayed by cells undergoing cell death [166,190]. Caspase activation, which is well-known to be involved in mediating the apoptotic cascade, were also observed in cryopreserved sperm cells [191–193]. The use of caspase inhibitors significantly improved the viability of hepatocytes and human embryonic stem cells after cryopreservation [194–196], indicating that preventing caspase activation can be a plausible approach to improve cell viability.

Some antioxidants may exert their effects through modulation of genes responsible for pro-survival, apoptosis and/or oxidative stress. Melatonin, iodixanol, catalase and vitamin E can up-regulate anti-apoptotic genes such as *Bcl2l1* (*Bcl-xL*) and *Bcl-2*, while down-regulating pro-apoptotic genes such as *BAX/Bax* [197–200]. With regard to melatonin, Deng et al. [199] and Chen et al. [198] observed different outcomes on *BAX/Bax* modulation. This difference could be attributed to the cell type used in each study. Apart from modulating genes responsible for cell survival, the pro-oxidative genes, *ROMO1* (in canine) and *NOX5* (in humans) have also been reported to be down-regulated by the administration of iodixanol [200] and melatonin, respectively [199]. The use of melatonin in cryopreservation has been noted to increase human antioxidant genes, such as *NRF2* and *SOD2* among others as shown in Table 1. It remains unclear if the up-regulation of antioxidant genes after antioxidant treatment provides direct benefit, if any, to protect cells against cryopreservation-induced ROS damage. Trehalose [201,202] and BHT [203] were reported to reduce lipid peroxidation [204] in testicular tissue and spermatozoa, respectively, generally enhance total antioxidant capacity, improve cellular viability [204–207] and reduce apoptosis [205,207]. Decrease in mitochondrial membrane potential (Δψ) has been observed in cells stimulated by apoptotic stimulus [208–211], which is also seen in thawed cells after cryopreservation [65]. The reduction in Δψ is, however, prevented through antioxidant administration [212–216]. Administration of amino acid with antioxidant properties such as l-proline is one such case where it reduces ROS levels as well as increases Δψ [217]. These studies indicate that supplementing antioxidants and/or factors that modulate the process of cell death can be a potential solution to reduce cryopreservation-induced cell death.

### Context-dependent effects of antioxidants in cryopreservation

The effectiveness of antioxidants in ameliorating functional parameters during cryopreservation is also dependent on the cell type used as well as the integrity of the cells prior to cryopreservation. This could be observed in the cryopreservation of sperm cells from different organisms. In one example, Dong et al. reported that the beneficial effects of SOD administration were only seen in sperm with low post-thaw survivability [76]. This is also observed for other antioxidants namely coenzyme Q [218], resveratrol [219], zinc sulfate [220], ascorbic acid [150], catalase [76] and 2,4-dinitrophenol [76]. These studies concluded that the effectiveness of antioxidants was dependent on sperm quality. While vitamins C and E may generally reduce DNA damage of spermatozoa from human and Gilt-head seabream (*Sparus aurata*) [148,149,151], these antioxidants can increase DNA damage in cryopreserved sperm from European seabass (*Dicentrarchus labrax*) [148] suggesting the possibility that antioxidants ameliorate freeze-thaw stress in a species-dependent manner. In cases where trehalose was used to cryopreserve spermatogonial stem cells, while the proliferation capability of such cells was increased *in vitro*, this did not translate to a real improvement in the number of colonies formed when such cells were subsequently transplanted [207]. Such disagreement between *in vitro* and *in vivo* results can also be seen when mouse embryos were incubated with ascorbic acid prior and post-cryopreservation, where the number of normal fetuses was unchanged despite notable improvements such as embryo intactness as well as blastocyst stage [183]. Consistently, there have also been reports in the literature where cellular functionality saw no improvement after antioxidant supplementation for cryopreservation [41,178,219,221,222]. Table 1 is a summary of the effect of antioxidants on cryopreserving reproductive cells and embryos.

### Potential practical applications of antioxidants and their effects on cellular function

Hemopoietic stem cells, hepatocytes and islet cells, all possess enormous potential when transplanted. In some studies, it has been reported that the ability to synthesize proteins [223] such as insulin [224,225] or albumin [226], metabolize xenobiotics [226,227], transplantation potential [226–228] and clonogenic potential [229–231] may either be lost or impaired via the process of cryopreservation. Such impairments or undesirable outcomes of cellular functionality have been partly improved through administration of antioxidants. These include ascorbic acid [221], astragalosides [232], taurine [222], hypotaurine [178], vitamin E [76], catalase [221], trehalose [205–207], combination of catalase with trehalose [233,234] as well as combination of BHT with ascorbic acid [235] to cell types such as mononuclear cells, pancreatic islets, germ cells, spermatozoa, dendritic cells, hepatocytes and hemopoietic cells (Table 1). Among these antioxidants, winter wheat lipocalins and peroxiredoxins obtained from wheat are especially notable. They were demonstrated to mollify cryopreservation-associated loss of attachment capacity of hepatocytes, as well as restoring the activity of CYP isoforms to the level similar from fresh, unfrozen murine hepatocytes [227,236]. Table 2 is a summary of the effect of antioxidants on non-reproductive cells.

Collectively, the different studies examined in this review indicated that the effectiveness of antioxidant supplement for cryopreservation very much depends on the cell type, organism as well as the specific antioxidant used. Table 1 provides a summary of the type of the antioxidant used, the cell type and organism as well as the effectiveness of the antioxidant based on the parameters measured.

## Moving forward

Oxidative stress is inevitably generated in the cryopreservation process and has been widely cited as the causative factor for some of the cryoinjuries inflicted on the cell [163,165,187,237]. Therefore, administering antioxidants in an effort to counter these deleterious effects on cells during cryopreservation is a plausible solution. Indeed, the use of antioxidants has undoubtedly conferred protection to certain cell type by improving several cellular function parameters and general cryopreservation outcome in specific circumstances as those indicated in the sections above and in Tables 1 and 2. Although effective in some circumstances, antioxidants can be ineffective or even deleterious for some cells. Antioxidants consist of a broad class of substances and molecules with varying physio-chemical properties that dictate their specificity, localization and/or ROS-scavenging roles [238,239]. Mitochondria-targeted antioxidants, MitoTEMPO [214] and melatonin [240,241] are potent antioxidants that prevent oxidative stress-associated damages encountered during cryopreservation. Melatonin in particular, has performed unexpectedly well by exerting its ROS-ameliorating properties through its multi-faceted mechanisms [242]. While the administration of melatonin improved the generation and survival of somatic cell nuclear transferred (SCNT) murine embryos from vitrified oocytes, whether melatonin directly affects ROS or inhibits apoptosis remains to be elucidated [243]. As such, the use of antioxidant in different combinations for the different cell types for cryopreservation may prove to be more effective in countering cryopreservation-induced ROS damage.

There are evidences to indicate that the use of different antioxidants in combination could provide additive protective effect when compared with those administered individually (Tables 1 and 2). In reproductive cells, catalase and low concentration of SOD have been reported to have no effect on oocyte survivability and fertility when used alone. However, when the same dose of SOD was co-administered with catalase, significant improvement in oocyte survivability was observed [124]. For the case of sperm cryopreservation, supplementation of SOD alone has no effect on the general sperm parameters such as motility (total and progressive motility), viability and percentage of sperm with high MMP, while supplementation of catalase alone was beneficial only at high concentrations [244]. Notably, when catalase and SOD were used in combination, sperm parameters such as total motility was greatly improved as compared with individual use of them at the respective dose [244]. In another example, sperm cells frozen in SOD and catalase, or vitamins C and E led to significantly improved parameters such as reduced ROS [215] and increased lateral head displacement of the sperm cells [245] whereas previously such parameters were not improved when the antioxidants were used individually [215,245].

Not all antioxidants used in combination yield additional benefits. For example, single administration of trehalose or catalase improved clonogenic parameters such as burst-forming unit erythroid and colony-forming unit granulocyte-monocyte in fetal liver hematopoietic cells and umbilical cord blood, respectively. However, when trehalose and catalase were administered in combination, no significant improvements in these parameters were observed [246]. Hence, use of different antioxidants in combination does not always imply additional improvement in cellular parameters.

Based on the studies examined in this review, it is notable that antioxidant supplement for cryopreservation can be effective. However, the effectiveness of the specific antioxidants depends on the cell type that undergoes the cryopreservation process. It is therefore important to consider supplementing cryopreservation media with specific antioxidants according to the specific species, cell type, quality and integrity of cells prior to cryopreservation. Additionally, several studies determined the efficacy of the antioxidants on cryopreservation by measuring functional parameters of the cryopreserved cells in an *ex vivo* setting. Given that many applications for cryopreservation are in the area of reproductive and regenerative medicine, future studies should be attempted to investigate the recovery and efficacy of the cryopreserved cells after transplantation *in vivo* to better understand the efficacy of the antioxidant used in this process.

## Abbreviations

ATF6: activating transcription factor 6
BHT: butylated hydroxytoluene
CPA: cryoprotective agent
CYP: cytochrome P450
DHE: dihydroethidium
DMSO: dimethyl sulfoxide
DUOX: dual oxidase
ER: endoplasmic reticulum
ERO: ER oxidoreductase
ETC: electron transport chain
GSH: glutathione
H _2_DCFDA: 2′,7′-dichlorodihydrofluorescein diacetate
H_2_O_2_: hydrogen peroxide
IRE1α: inositol-requiring enzyme 1 α
l-NAME: NG-nitro-l-arginine methyl ester
MDA: malondialdehyde
MMP: mitochondrial membrane potential
mPTP: mitochondria permeability transition pore
NOS: nitric oxide synthase
NOX: NADPH oxidase
PBMC: peripheral blood mononuclear cell
PDI: protein disulfide isomerase
PERK: pancreatic eIF-2alpha kinase
ROS: reactive oxygen species
SAB: SH3 homology associated BTK binding protein 5
SOD: superoxide dismutase
TUDCA: tauroursodeoxycholic acid
UPR: unfolded protein response
XBP-1: X-box binding protein 1
4-HNE: 4-Hydroxynonenal
γH2AX: H2A histone family member X
